# Publisher Correction: Critical Southern Ocean climate model biases traced to atmospheric model cloud errors

**DOI:** 10.1038/s41467-018-06662-8

**Published:** 2018-10-02

**Authors:** Patrick Hyder, John M. Edwards, Richard P. Allan, Helene T. Hewitt, Thomas J. Bracegirdle, Jonathan M. Gregory, Richard A. Wood, Andrew J. S. Meijers, Jane Mulcahy, Paul Field, Kalli Furtado, Alejandro Bodas-Salcedo, Keith D. Williams, Dan Copsey, Simon A. Josey, Chunlei Liu, Chris D. Roberts, Claudio Sanchez, Jeff Ridley, Livia Thorpe, Steven C. Hardiman, Michael Mayer, David I. Berry, Stephen E. Belcher

**Affiliations:** 1 Met Office Hadley Centre, FitzRoy Road, Exeter, UK; 2 Department of Meteorology, University of Reading, PO Box 243, Whiteknights Campus Earley Gate, Reading, RG6 6BB UK; 3 National Centre for Earth Observations (NCEO), University of Reading, PO Box 243, Whiteknights Campus Earley Gate, Reading, RG6 6BB UK; 4 British Antarctic Survey, High Cross, Madingley Road, Cambridge, CB3 0ET UK; 5 National Centre for Atmospheric Sciences (NCAS), University of Reading, PO Box 243, Whiteknights Campus Earley Gate, Reading, RG6 6BB UK; 6 School of Earth and Environment, University of Leeds, Leeds, LS2 9JT UK; 7 National Oceanography Centre, European Way, Southampton, SO14 3ZH UK; 8 Department of Meteorology and Geophysics, University of Vienna, Althanstraße 14, Vienna, 1090 Austria

Correction to: *Nature Communications*; 10.1038/s41467-018-05634-2; published online 11 September 2018

In the original HTML version of this Article, ref. 12 was incorrectly cited in the first sentence of the first paragraph of the Introduction. The correct citation is ref. 2. This has now been corrected in the HTML version of the Article; the PDF version was correct at the time of publication.

