# Supplementary material for: Critical Southern Ocean climate model biases traced to atmospheric model cloud errors
Source: Nat Commun. 2018 Sep 11;9:3625. doi: 10.1038/s41467-018-05634-2 (PMC6134029; doi:10.1038/s41467-018-05634-2)
Supplement: Supplementary file 1 — Supplementary Information [file 41467_2018_5634_MOESM1_ESM.pdf]

Critical Southern Ocean climate model biases traced to atmospheric model cloud errors.

Patrick Hyder*, John M. Edwards, Richard P. Allan, Helene T. Hewitt, Thomas J. Bracegirdle, Jonathan M. Gregory, Richard A. Wood, Andrew J. S. Meijers, Jane Mulcahy, Paul Field, Kalli Furtado, Alejandro Bodas-Salcedo, Keith D. Williams, Dan Copsey, Simon A. Josey, Chunlei Liu, Chris D. Roberts, Claudio Sanchez, Jeff Ridley, Livia Thorpe, Steven C. Hardiman, Michael Mayer, David I. Berry and Stephen E. Belcher

* Correspondence to patrick.hyder@metoffice.gov.uk

ARTICLE FOR NATURE COMMUNICATIONS

SUPPLEMENTARY INFORMATION includes:
(1) Supplementary tables
(2) Supplementary figures
(3) Supplementary references

(1) Supplementary Tables

AMIP5 model names	CMIP5 model names	CMIP5 SST & heat content bias on AMIP5 net flux bias regressions	CMIP5 ZWML bias on AMIP5 net flux bias regression	AMIP5 ZWML bias on AMIP5 net flux bias regression	CMIP5 ZWML bias on CMIP5 SST bias regression	SST on AMOC strength regression
ACCESS1-0	ACCESS1-0	X	X	X	X	
ACCESS1-3	ACCESS1-3	X	X	X	X	X
bcc-csm1-1-m	bcc-csm1-1-m	X	X		X	
BNU-ESM	BNU-ESM	X	X	X	X	
CanAM4	CanESM2	X				X
CCSM4	CCSM4	X				
CESM1-CAM5	CESM1-CAM5	X				
CMCC-CM	CMCC-CM	X	X	X	X	
CNRM-CM5	CNRM-CM5	X	X	X	X	X
CSIRO-Mk3-6-0	CSIRO-Mk3-6-0	X	X	X	X	X
FGOALS-g2	N/A					X
FGOALS-s2	N/A					
GFDL-CM3	GFDL-CM3	X	X		X	X
N/A	GFDL-ESM2M					X
GFDL-HIRAM-C180	N/A					
GFDL-HIRAM-C360	N/A					
GISS-E2-R	GISS-E2-R	X	X	X	X	
HadGEM2-A	HADGEM2-ES	X	X		X	X
inmcm4	Inmcm4		X	X		
IPSL-CM5A-LR	IPSL-CM5A-LR	X	X	X	X	
IPSL-CM5A-MR	IPSL-CM5A-MR	X	X	X	X	
MIROC5	N/A		X	X		
MPI-ESM-LR	MPI-ESM-LR	X	X	X	X	X
MPI-ESM-MR	MPI-ESM-MR	X	X	X	X	X
N/A	MPI-ESM-P					X
MRI-AGCM3-2H	MRI-CGCM3		X	X		
MRI-AGCM3-2S	N/A					
MRI-CGCM3	N/A					
NorESM1-M	NorESM1-M	X	X	X	X	X
N/A	NORESM1-ME					X
No. of models		18 *	18	15	15	13

Supplementary Table 1 AMIP5 and CMIP5 models used for the regression and atmospheric heat flux analyses. ZWML is Zonal Wind Maximum Latitude and AMOC is Atlantic Meridional Overturning Circulation. * These 18 models with consistent CMIP5 and AMIP5 experiments were used for the multi-model means and standard deviations, and for the AMIP5/CMIP5 net flux, flux component and SST bias regression analyses. Details of model configuration for most of these models are included in Table 9.A.1 of Flato *et al.* (2013). Additional information on the configuration of the HadGEM2-ES model is presented in Jones *et al.* (2011).

(2) Supplementary figures

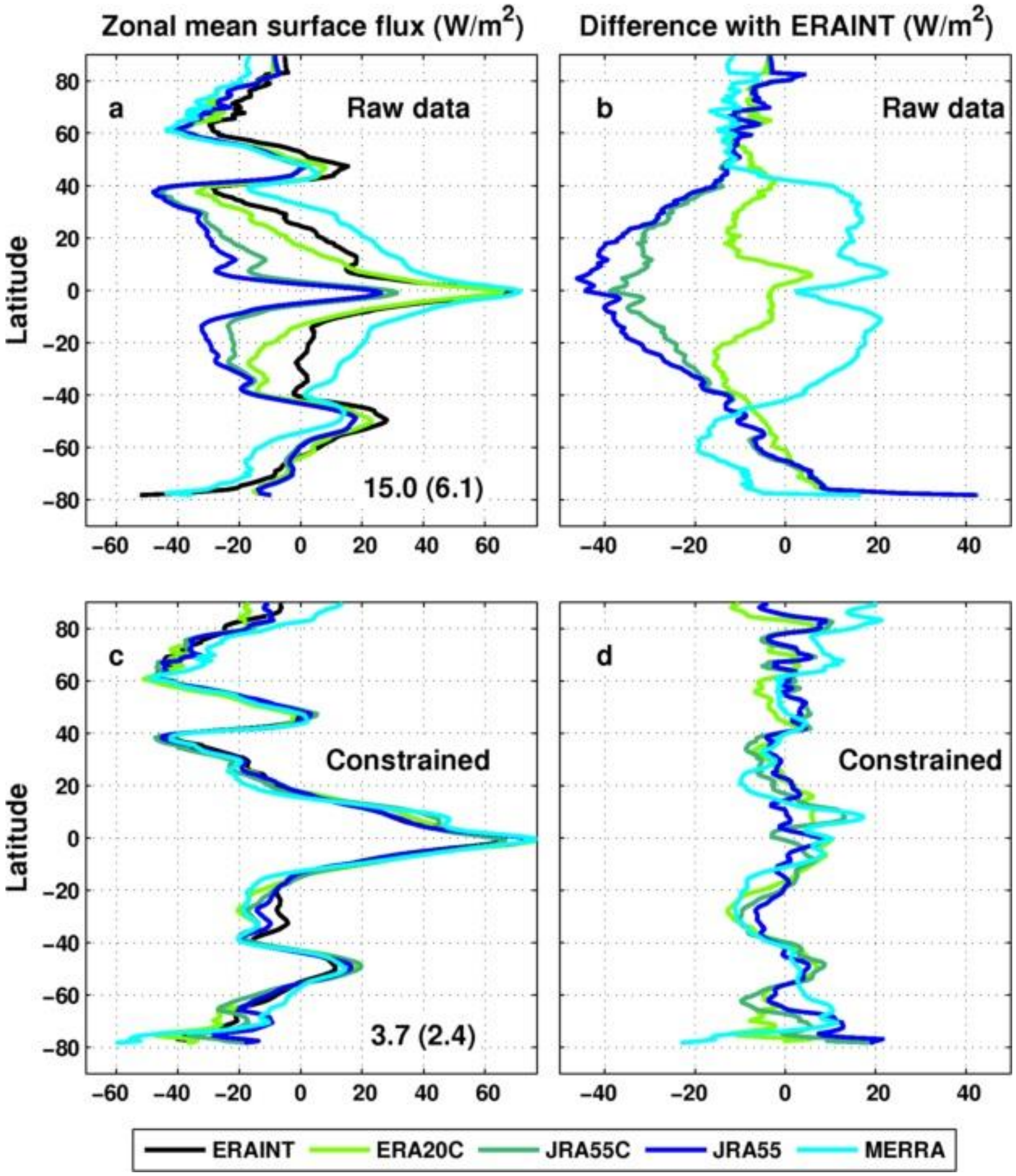

Supplementary Figure 1 Zonal mean net surface downward ocean fluxes from several atmospheric reanalyses (2001-2007 averages). (**a**) direct reanalysis model flux diagnostics, (**b**) differences in direct reanalysis flux diagnostics from those for ERA-Interim, (**c**) fluxes estimated using our method employing atmospheric column energy conservation and (**d**) the differences in fluxes using our method from those for ERA-Interim**.** The means across all latitudes and for the 40-60°S Southern Ocean (in brackets) of zonal mean standard deviations across the models are displayed in (**a**) and (**c**).

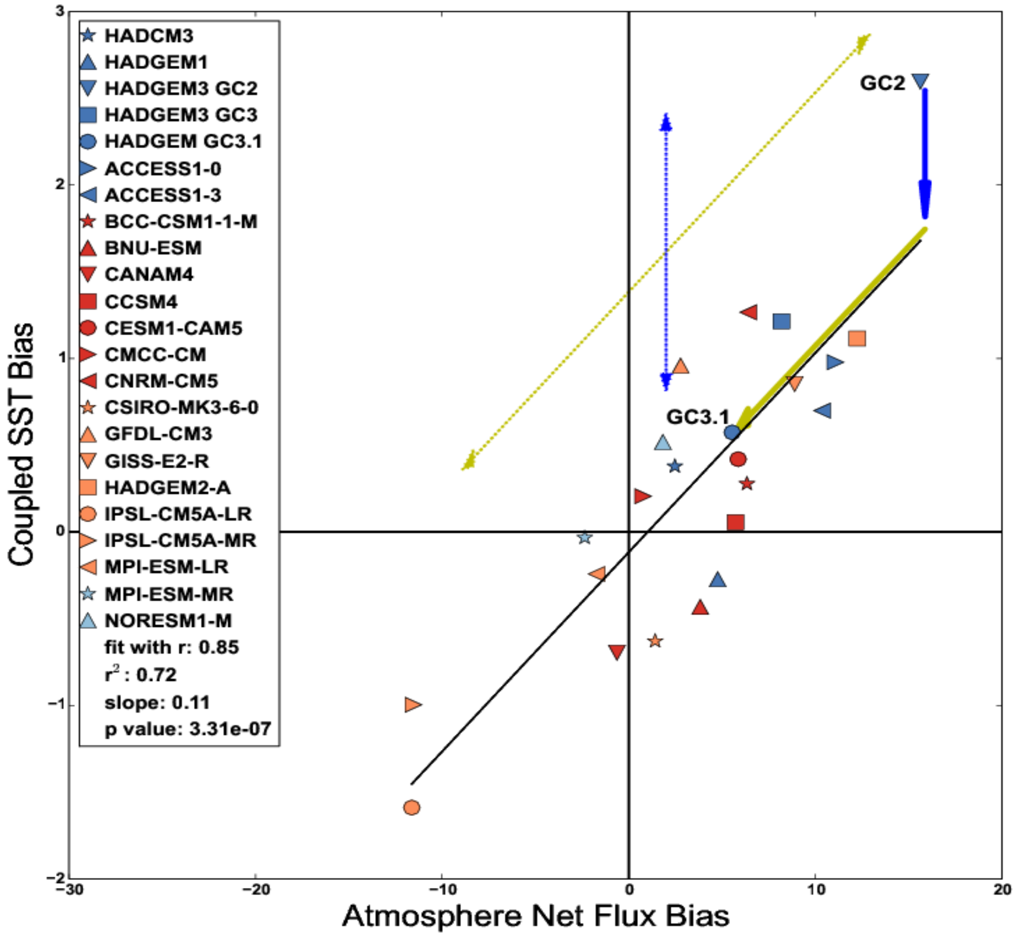

Supplementary Figure 2 Linear regression of the estimated CMIP5 and Hadley Centre coupled model SST biases on the estimated AMIP5 and Hadley Centre atmospheric model net surface downward heat flux biases. Stand-alone atmospheric net flux bias changes associated with model developments could be expected to move models approximately parallel to regression line (yellow dotted arrows). If atmospheric net flux biases are unchanged, changes to other drivers of SST biases will move models vertically (blue dotted arrows). Such drivers include ocean model errors, and atmospheric model momentum or freshwater forcing errors. The improvements from HadGEM3-GC2 (N216-1/4°) to HadGEM3-GC3.1 (N216-1/4°) include a contribution from ocean model improvements (thick solid blue arrow) and atmospheric net flux improvements (thick solid yellow arrow). Between GC2 and GC3.1 the configuration of the ocean model vertical mixing was changed, which substantially improved the shallow mixed layer depths, and the along-isopycnal mixing coefficient was reduced. The HadGEM3-GC2 and HadGEM3-GC3.1 SST biases are for present day control runs for years 50 to 100 whilst the other Hadley centre model and CMIP5 SST biases are for the historical experiment (see Methods).

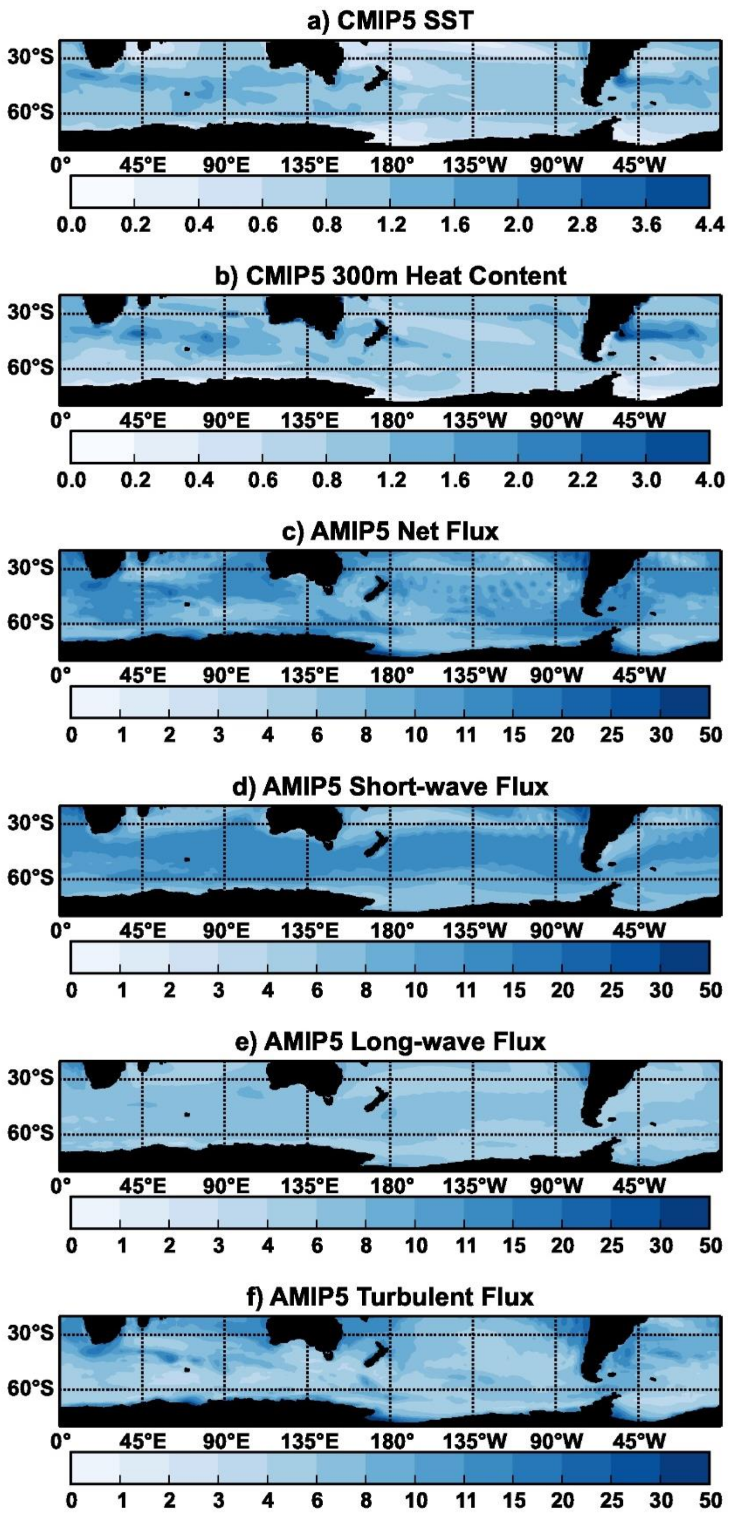

Supplementary Figure 3 Standard deviations across the model ensemble in CMIP5 SST and heat content and AMIP5 surface heat flux components. (**a**) CMIP5 SST bias (^{o}C), (**b**) CMIP5 upper ocean 300m heat content bias, expressed as a layer mean temperature (^{o}C), (**c**) AMIP5 net downwards surface flux bias (Wm^{-2}), (**d**) AMIP5 net surface short-wave radiation flux bias (Wm^{-2}), (**e**) AMIP5 net surface long-wave radiation flux bias (Wm^{-2}), and (**f**) AMIP5 net downward surface total turbulent heat flux bias (Wm^{-2}).

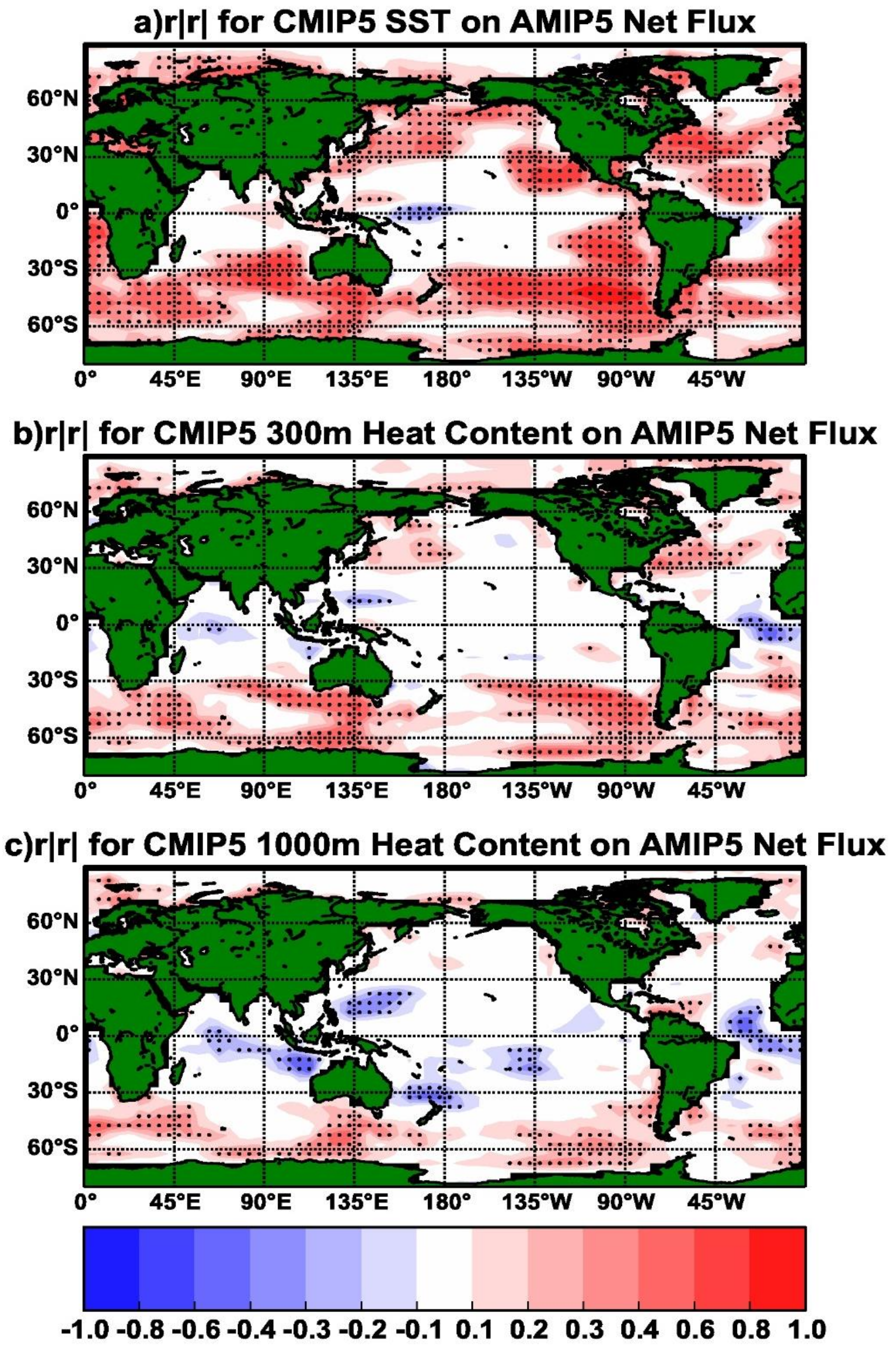

Supplementary Figure 4 Fraction of explained variance for regressions of CMIP5 SST, 300m heat content and 1000m heat content biases on AMIP5 net flux biases. Linear regressions were undertaken spatially on a 5x5^o grid for (**a**) estimated CMIP5 SST biases, (**b**) estimated CMIP5 upper 300m heat content biases, and (**c**) estimated CMIP5 upper 1000m heat content biases, on estimated AMIP5 net surface downward flux biases. Fraction of explained variance is presented as r|r|, to retain the sign of the correlation, r. Regions where the p-values (see Methods) for the correlation are less than 0.05 are stippled. Heat contents are expressed as layer mean temperatures (K).

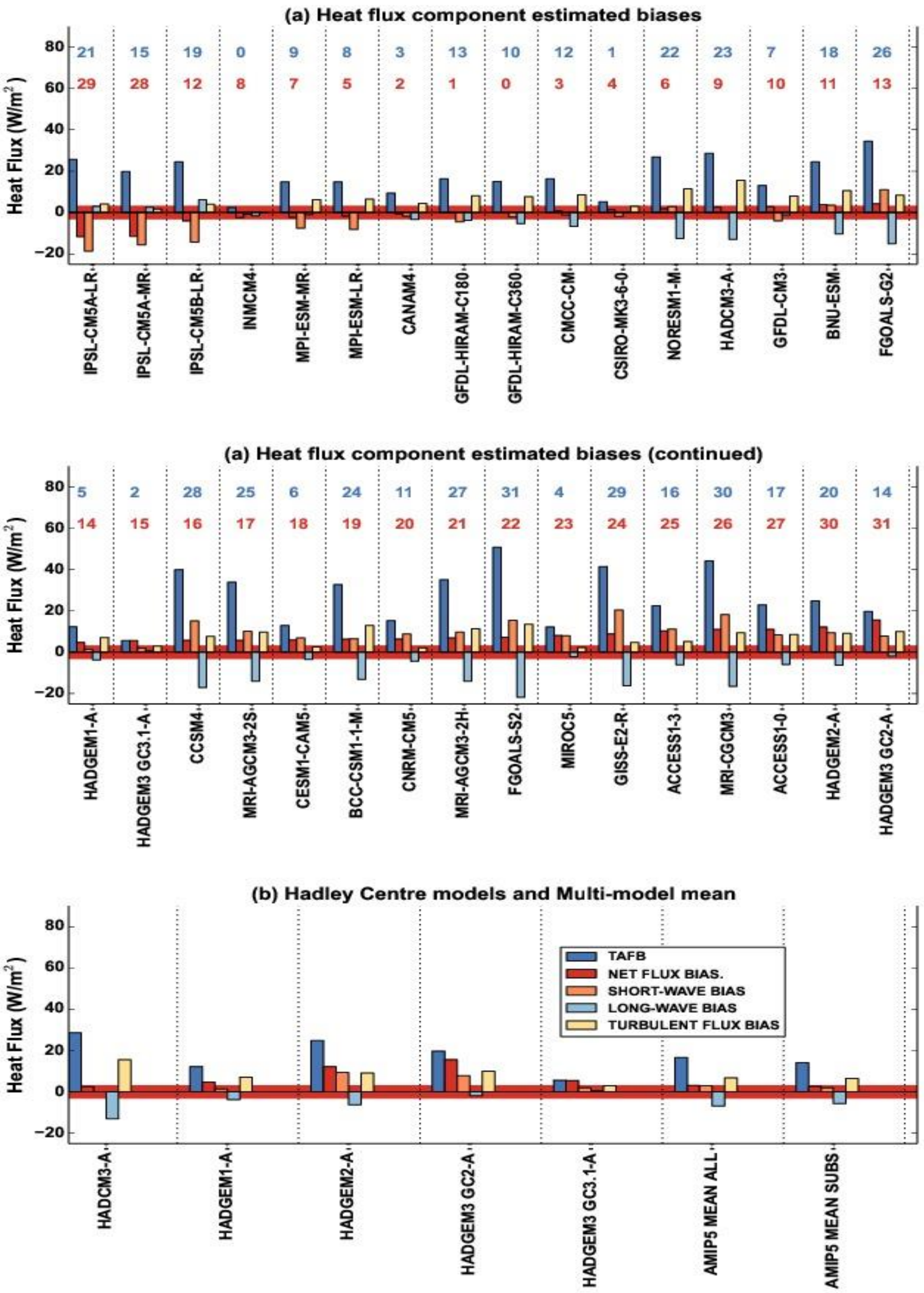

Supplementary Figure 5 Bar chart presenting 40-60°S surface heat flux biases for the AMIP5 and Hadley Centre atmospheric models. Estimated Total Absolute Flux Biases (TAFB) are presented together with biases in simulated net downward heat flux, short-wave radiation flux, long-wave radiation flux and total turbulent heat flux (continued on next page).

Supplementary Figure 5 (continued) In panels (a) the AMIP5 models and Hadley Centre atmospheric models are plotted in order of increasing estimated net flux biases from left to right. The indicative model rank numbers, from 0 (for best) to 31 (worst) are labelled based on the absolute magnitude of net flux bias (in red) and Total Absolute Flux Biases (TAFB) (in dark blue). In panel (b) the succession of Hadley Centre models is plotted in order of release date. Also included is the multi-model AMIP5 mean flux components and the TAFB derived from these means for all AMIP5 models (AMIP5 MEAN ALL) and for the subset of 18 AMIP5 models with CMIP5 SST diagnostics (AMIP5 MEAN SUBS). Bias variations between models do not depend on the observations. However, individual model bias estimates include a contribution from observational errors (which would change these rank numbers). Indicative estimates of these observational errors are ~± 3, 1, 6, 7 and 8Wm^{-2} for net downward total flux, surface short-wave, long-wave, total turbulent and TAFB, respectively (see Methods). Estimated biases or TAFB remain uncertain and should be interpreted with caution, if their values are of comparable or smaller magnitude than the observational uncertainties. Red shading in panel (a) and (b) represents our estimate of ±3Wm^{-2} uncertainty for a zero net flux bias. The mean observational estimates for 40-60°S for net, short-wave, long-wave and total turbulent fluxes are 1, 116, -40 and -75Wm^{-2}, respectively. To provide an estimate of fractional size of simulated TAFB, the sum of observed absolute short-wave, long-wave and total turbulent heat fluxes is 230Wm^{-2}.

Supplementary References

Flato G. J. *et al*. Evaluation of Climate Models. In: *Climate Change 2013: The Physical Science Basis. Contribution of Working Group I to the Fifth Assessment Report of the Intergovernmental Panel on Climate Change*, Cambridge University Press, UK (2013).

Jones, C. D. *et al*. The HadGEM2-ES implementation of CMIP5 centennial simulations, *Geosci. Model Dev*., **4**, 543–570 (2011).
